# Long-Branch Attraction Bias and Inconsistency in Bayesian Phylogenetics

**DOI:** 10.1371/journal.pone.0007891

**Published:** 2009-12-09

**Authors:** Bryan Kolaczkowski, Joseph W. Thornton

**Affiliations:** 1 Center for Ecology and Evolutionary Biology, University of Oregon, Eugene, Oregon, United States of America; 2 Howard Hughes Medical Institute, University of Oregon, Eugene, Oregon, United States of America; University of California San Diego, United States of America

## Abstract

Bayesian inference (BI) of phylogenetic relationships uses the same probabilistic models of evolution as its precursor maximum likelihood (ML), so BI has generally been assumed to share ML's desirable statistical properties, such as largely unbiased inference of topology given an accurate model and increasingly reliable inferences as the amount of data increases. Here we show that BI, unlike ML, is biased in favor of topologies that group long branches together, even when the true model and prior distributions of evolutionary parameters over a group of phylogenies are known. Using experimental simulation studies and numerical and mathematical analyses, we show that this bias becomes more severe as more data are analyzed, causing BI to infer an incorrect tree as the maximum a posteriori phylogeny with asymptotically high support as sequence length approaches infinity. BI's long branch attraction bias is relatively weak when the true model is simple but becomes pronounced when sequence sites evolve heterogeneously, even when this complexity is incorporated in the model. This bias—which is apparent under both controlled simulation conditions and in analyses of empirical sequence data—also makes BI less efficient and less robust to the use of an incorrect evolutionary model than ML. Surprisingly, BI's bias is caused by one of the method's stated advantages—that it incorporates uncertainty about branch lengths by integrating over a distribution of possible values instead of estimating them from the data, as ML does. Our findings suggest that trees inferred using BI should be interpreted with caution and that ML may be a more reliable framework for modern phylogenetic analysis.

## Introduction

Statistical inference of phylogenetic relationships informs analysis in fields as diverse as comparative genomics, epidemiology, ecology, and evolution [1]. Bayesian inference (BI) of phylogeny [2]–[4] has recently gained in popularity and appears to have answered some long-standing phylogenetic questions [5], [6]. The aim of Bayesian statistics is typically to characterize the posterior probability distribution of a set of hypotheses, given a body of data, a probabilistic model for the generation of that data, and an explicit probabilistic description of prior beliefs. The chief concern of phylogenetics, in contrast, is to produce a concrete inference of historical evolutionary relationships and to characterize the statistical support for that inference. As such, nearly all phylogenetic analyses using BI have applied a Bayesian decision rule to select the tree with the highest posterior probability (or a consensus tree of all clades with posterior probability 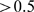) as the best hypothesis of phylogeny (e.g., [5], [6]).

BI and its precursor maximum likelihood (ML) infer phylogenetic relationships using the same probabilistic models of molecular evolution, so it has been assumed that BI, like ML [7]–[9], is largely unbiased and statistically consistent given the correct model [6], [10]. A key difference between BI and ML—and a major proposed advantage of BI [3], [10]–[12]—is that Bayesian methods incorporate uncertainty about “nuisance parameters” such as branch lengths on the topology and the parameters of the evolutionary model; in contrast, ML requires specific values for these parameters to be estimated from the data. When data are limited, the ML estimates may deviate from the true values, because the observed state pattern frequencies vary stochastically from expectation. With larger datasets, ML yields increasingly accurate estimates of nuisance parameter values; as sequence length approaches infinity, the likelihood of the true phylogeny (with the correctly estimated branch lengths) is guaranteed to exceed that of any other phylogeny (with any branch lengths), so long as the model is adequately parameterized and identifiable [9], [13]. In order to reduce dependence on estimates of nuisance parameters, BI calculates the integrated likelihood of each topology over multiple values of each parameter, weighted by a user-specified distribution that describes the prior probability of each parameter value [3]. Reliable prior information about branch lengths and other model parameters is seldom available in practice, so virtually all analyses have used “uninformative” diffuse prior distributions (such as branch length priors uniform from 0 to 5 or exponential with mean 0.1, which are offered as the default values in common software packages). Because BI incorporates uncertainty about nuisance parameters, it has been favored over ML for implementing complex models with many parameters, particularly when data are limited [3], [10], [12], [14].

The statistical characteristics and performance of BI, particularly vis-a-vis ML, have not been thoroughly evaluated. Several criteria can be used to evaluate the reliability of phylogenetic methods for inferring topologies. First, the asymptotic performance of phylogenetic methods when the assumed evolutionary model is correct has been evaluated in terms of statistical consistency—convergence in probability on the true phylogeny, typically with increasing support, as the amount of sequence data increases. Consistency has been evaluated directly by mathematical proof [9], [15]–[18] or numerical analysis [19], [20], and indirectly by analyzing simulated datasets of increasing size [21]–[23]. Second, topological bias has been evaluated by determining whether a method tends to recover a particular incorrect topology when phylogenetic signal is absent or weak [7], [19], [24]. Third, efficiency—the quantity of data required to reliably recover the true tree—has typically been assessed by analyzing the proportion of correct inferences using simulated datasets of variable size [25]–[27]. Fourth, robustness to incorrect assumptions about the underlying evolutionary model or incorrect prior distributions—an important practical concern, because complete and accurate a priori knowledge of evolutionary processes is never available—has been evaluated by examining consistency, bias, and efficiency when the true model and prior distributions are not applied [8], [23], [28]–[32]. Other studies have examined the accuracy and behavior of measures of statistical confidence in topological inferences [29], [32]–[40].

Most analyses of Bayesian phylogenetic methods have focused on the properties of its confidence measures; the consistency, bias, efficiency, and robustness of using BI with a Bayes decision rule to infer topologies have not been well characterized. “Bayesian simulations” have shown that, when the prior distributions precisely match the distribution of conditions under which the data were simulated, the average posterior probability of a group of inferences accurately predicts the proportion of those inferences that are correct [29], [31]. Yang and Rannala [31] showed that the choice of priors affects posterior probabilities and that vague or uninformative priors can cause them to deviate from the fraction of correct inferences, but they did not investigate whether the deviation was structured to favor certain topologies. Kolaczkowski and Thornton [32] found that the direction of this deviation in posterior probabilities depends on the pattern of branch lengths on the tree; when the true tree has non-sister long branches, the posterior probability of the incorrect long branch attraction (LBA) tree tends to be inflated. Susko [41] analyzed the distribution of posterior probabilities in the limiting case of sequence length approaching infinity and found that sequences generated on an unresolved four-taxon star tree with two long branches yield posterior probabilities that favor the resolved LBA tree. Taken together, these studies establish that the choice of prior distribution affects posterior probabilities and suggest that under some simple conditions BI might exhibit topological bias.

Many questions remain open, however. First, it is not clear whether BI using a Bayesian decision rule is significantly biased when finite data are analyzed, when the true tree is resolved, or when sequences generated under realistic conditions are analyzed. Second, it is unclear why BI might be biased in favor of certain topologies as data increases, particularly because the effects of prior assumptions are expected to diminish as the quantity of data increases. Third, the possibility that Bayesian simulations—in which results are summarized over a range of evolutionary conditions—might mask bias under specific conditions has not been examined. Finally, the relative accuracy, efficiency, and robustness of BI compared to ML has not been evaluated.

BI and ML implementations employ different search strategies and different estimates of statistical confidence, so direct comparison of phylogenetic accuracy using these two frameworks has not been possible. To address this issue, we implemented a novel “empirical Bayes” [42] method, which uses the same Markov-chain Monte Carlo (MCMC) sampling strategy as traditional BI but calculates the posterior probability of each tree assuming the ML estimate of branch lengths and other parameters (Fig. S1). Although posterior probabilities are not a meaningful concept in a strict ML framework, our empirical Bayes approach produces inferences identical to those generated by ML: given uniform prior probability for each topology and an adequate search, the tree with the highest posterior probability using our method will always be the ML tree. BI differs from our ML/empirical Bayes method only by integrating over branch lengths and other model parameters, allowing us to directly compare the performance of ML to BI and to specifically determine the effects of incorporating parameter uncertainty on phylogenetic accuracy.

We analyzed both simulated and empirical data under a range of controlled conditions using both BI and this novel ML implementation. The results, together with numerical and mathematical analyses, indicate that integrating over uncertainty about branch lengths induces an intractable topological bias in BI that results in reduced accuracy, efficiency, and robustness compared to ML; they also suggest that BI is likely to be statistically inconsistent. Although in practice BI and ML will recover the same phylogeny across a wide range of conditions, our findings indicate that when the two methods differ in their results, ML is more likely to be accurate.

## Results

### Long Branch Attraction Bias

**Figure 1:**
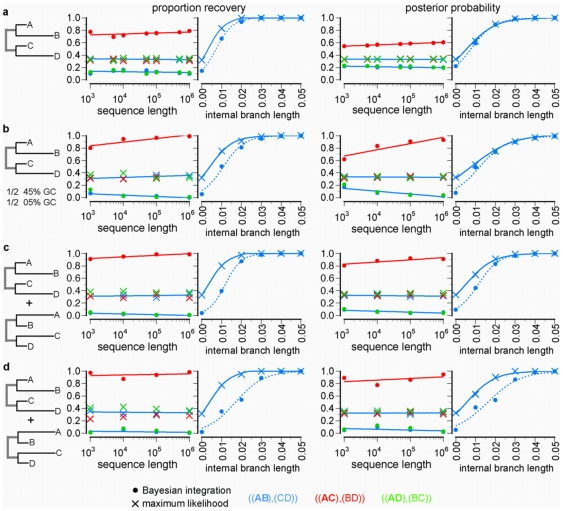
Maximum likelihood overcomes long branch attraction bias caused by integrating over parameter uncertainty. Nucleotide sequences (500 replicates) of increasing length were generated on the topology shown with two long (0.75 substitutions/site) and two short (0.05) terminal branches and a variable internal branch. In each row, the left two panels show the proportion of replicates from which each resolved topology was inferred, plotted against increasing sequence length (left) or internal branch length on the true tree (right). The right two panels plot the mean posterior probability over replicates of each resolved topology. For plots over increasing sequence length, the internal branch length was fixed at zero. The true evolutionary model was used in all analyses. **a**, Sequences were generated using a simple model with no heterogeneity. **b**, Half the sites evolved with elevated G+C content (45%), and the other half had reduced G+C (5%); data were analyzed using a correctly partitioned model. **c**, Sequences were generated under a heterotachous model in which half the sites evolved along a tree with long terminals to B and D, while the other half had taxa A and C with long branches; data were analyzed using a correctly partitioned model. **d**, Sequences were generated under the same heterotachous model as in **c** and analyzed with a two-class heterotachous mixture model.

We first evaluated whether incorporating parameter uncertainty using BI as commonly practiced causes topological bias under simple but challenging evolutionary conditions [19]. We simulated sequences using a simple model of nucleotide evolution along a four-taxon star tree with two long and two short terminal branch lengths (Fig. 1a). When data were analyzed using the correct evolutionary model, ML was unbiased, recovering each possible tree with equal frequency; the mean posterior probability for each tree was ∼1/3 at all sequence lengths, as expected for an unbiased method [24]. In contrast, BI—using the common assumption of uniform priors over branch lengths—inferred as the maximum a posteriori tree the falsely resolved topology that pairs long branches together from over 70% of replicates, with mean posterior probability ∼0.6, when sequences were of moderate length. This long branch attraction (LBA) bias grew stronger with increasing sequence length, as indicated by a positive slope of the best-fit regression curve (*P* = 0.03). BI's bias is not restricted to star-tree conditions but affects phylogenetic accuracy on resolved trees, as well (Fig. 1a). Under simple evolutionary conditions, BI required a 25% longer internal branch than ML to recover the correct phylogeny with 95% frequency (Table S1). These results indicate that BI suffers from long branch attraction bias and that this bias is caused by integrating over branch lengths. They also establish that, under these conditions, BI is less efficient than ML at recovering the true topology.

We conducted similar analyses using both nucleotide and amino acid data, various prior distributions, and a range of complex and simple evolutionary models. In all cases, BI—unlike ML—displayed LBA bias, which grew worse with increasing data (Figs. S2, S3). The bias persisted when exponential priors and any other available distributions in MrBayes [43] were used, and some prior distributions greatly exacerbated the bias (Fig. S4). Novel priors that apply distinct exponential distributions to internal and terminal branch lengths [31] did not eliminate long branch attraction (). It has been suggested that the failure of existing MCMC algorithms to explicitly sample zero-length branches could produce unreliable results when the true tree is a star phylogeny [30], but BI remained biased when modified to sample unresolved trees (Fig. S5b,c).

### Increasing Bias with More Complex Models

A second proposed advantage of BI over ML is that Bayesian MCMC provides a more reliable method for analyzing data using complex models that incorporate evolutionary heterogeneity among sites, such as those that use mixture models or partition sites into independent classes [3], [10], [12], [14]. To determine the effect of integrating over uncertainty when sites in a sequence evolve and are analyzed under complex heterogeneous models, we simulated sequences with strong across-site heterogeneity in G+C content or site-specific changes in evolutionary rates (heterotachy, represented as different branch length sets for different sites [44]). When these data were analyzed using the correct partitioned and mixture models, BI's bias became considerably more severe than on homogeneous sequences, with the LBA tree being recovered from nearly 100% of replicates (Fig. 1b,c,d). In each case, adding more data increased the intensity of the bias (*P* 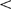
0.001), and the posterior probability of the incorrect tree converged to 1.0. ML, in contrast, remained unbiased in all these analyses. Using more complex models also exacerbated the performance difference between BI and ML on resolved trees (Fig. 1b,c,d). For example, when the mixture model was used to incorporate heterotachy, BI required twice as long an internal branch to achieve the same accuracy as ML (Table S1). These results show that using complex models that integrate over many nuisance parameters causes BI's intrinsic long branch attraction bias to become more severe.

### Increased Sensitivity to Model Violations

**Figure 2:**
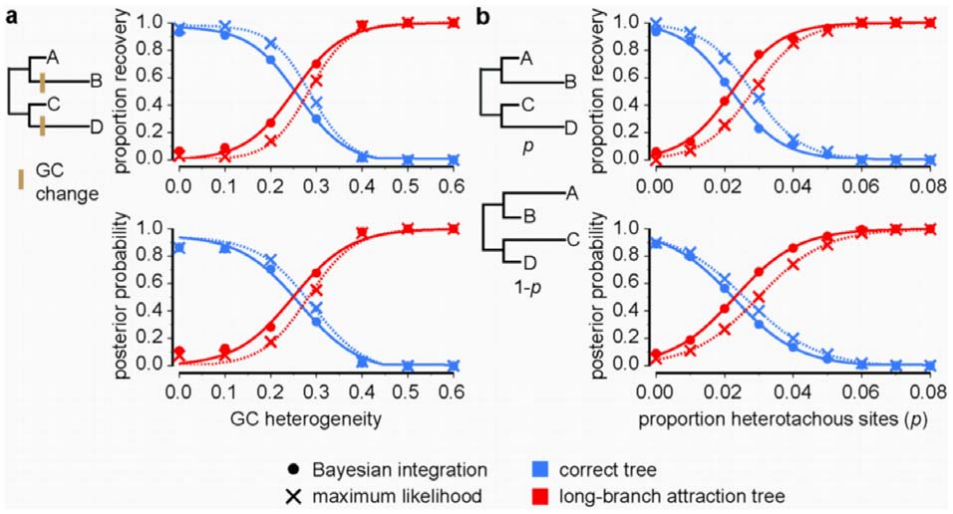
ML is less susceptible than BI to long branch attraction caused by model violations. Datasets of 5,000 nt were generated using heterogeneous evolutionary models on a four-taxon tree with non-sister long terminal (0.75 substitutions/site) and short terminal (0.05) branch lengths and an internal branch of 0.02, then analyzed using a simple homogeneous model. We plotted the proportion of replicates from which each topology was recovered, as well as the mean posterior probability of each tree, as evolutionary heterogeneity increased. **a**, Sequences were generated with convergent G+C content in non-sister lineages. GC heterogeneity indicates absolute increase of G+C content in the marked lineages above ancestral baseline of 30%. **b**, Two classes of heterotachous sites evolved on the same topology but with different branch lengths for each class. We varied the strength of heterogeneity by increasing from zero the proportion of sites in the first class.

Statistical models used to infer phylogenies are simplifications of the real evolutionary process, so analyses of real data are always conducted using a too-simple model. To determine the relative sensitivity of BI and ML to model violation, we simulated sequences with two types of common model violation—heterotachy and lineage-specific changes in G+C content—on a resolved four-taxon tree with two long branches; we then analyzed these data assuming common homogeneous models (Fig. 2). When heterogeneity was weak, both BI and ML recovered the correct tree with strong support; when heterogeneity of either type was strong, both methods were biased in favor of the LBA tree. Between these extremes, ML recovered the correct phylogeny significantly more often than BI (*P* 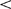
0.001), indicating that BI is more sensitive to model violations. Although the advantage of ML over BI was never greater than ∼20%, we found regions of parameter space in which ML strongly supports the correct tree, while BI supports the LBA tree. In still other regions, BI strongly supports the incorrect LBA tree, while ML is only weakly biased.

### Bias under Empirical Conditions

**Figure 3:**
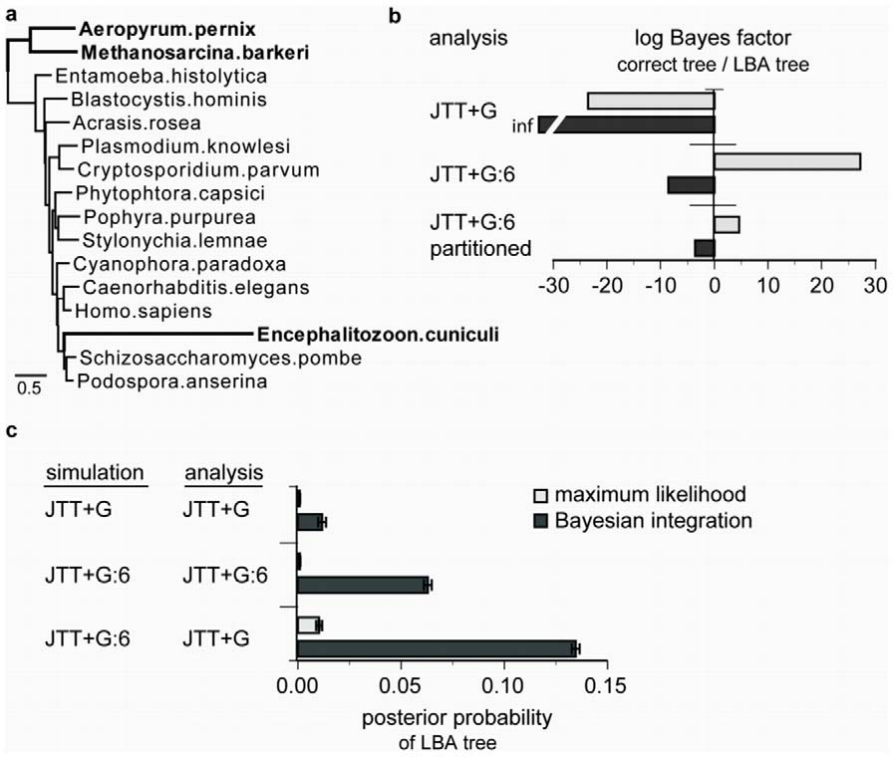
ML is less susceptible than BI to long branch attraction under empirical conditions. **a**, The correct eukaryote phylogeny places the microsporidian *Encephalitozoon cuniculi* with the fungi, as shown. The long branch attraction (LBA) tree pairs taxa in bold. **b**, We analyzed elongation factor-1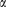 data using three evolutionary models: 1) JTT+G, Jones-Taylor-Thornton model of amino acid replacements with gamma-distributed among-site rate variation; 2) JTT+G:6, heterotachous mixture model with 6 branch-length classes, and 3) a 6-category partitioned model, with partitions inferred using JTT+G:6. For each model, we plot the log Bayes factor of the correct placement of microsporidia vs. the LBA tree, with positive values indicating support for the correct phylogeny (see Methods). Label ‘inf’ indicates maximal support for the LBA clade; the correct tree was not sampled during the MCMC run. **c**, We simulated 200 replicate sequence alignments of 500 residues along the tree in panel **a**, with branch lengths and model parameters estimated from elongation factor-1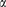 data. Models used to simulate and analyze datasets are indicated in the figure. For each combination of models, we plotted the posterior probability of the incorrect LBA clade; bars indicate standard error.

The results reported above establish that BI produces biased inferences under extreme conditions on small trees. To determine the relative performance of BI and ML when larger phylogenetic problems and real molecular sequence data are analyzed, we examined a well-known case of phylogenetic error (Fig. 3a). When eukaryote elongation factor-1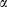 (EF1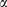) sequences are analyzed using traditional ML and BI, the microsporidian *Encephalitozoon cuniculi* is artifactually attracted to the long branch leading to the archaebacterial outgroup (the MA tree), instead of its correct placement with fungi (the MF tree) [45], [46]; previous work has shown that unincorporated heterotachy contributes to this error [45], [47]. When we analyzed the empirical EF1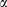 data using a homogeneous model, both ML and BI favored the incorrect MA tree, but the support for the incorrect tree was much stronger with BI than ML (Fig. 3b). When a mixture model was used to incorporate heterotachy, BI continued to prefer the incorrect MA tree, but ML recovered the true tree with strong support. When the data were analyzed using a partitioned model that groups sites according to rate classes inferred by the mixture model, ML recovered the true tree, whereas BI continued to be biased in favor of the MA tree. Under realistic conditions, ML is therefore less susceptible to long branch attraction than BI, and complex models—both mixed and partitioned—perform better in an ML than a BI framework.

To determine the relative contributions of intrinsic bias, model complexity, and model violation to BI's poor performance in this case, we simulated protein sequences of 500 residues along the eukaryote phylogeny with branch lengths and model parameters estimated from the empirical EF1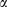 data. We found that all three factors contribute. When data were simulated and analyzed under a homotachous rates model, ML showed no support for the incorrect MA phylogeny, whereas BI did support this tree, albeit weakly (Fig. 3c). When data were simulated using a heterotachous mixture model with parameters derived from the empirical data and then analyzed using the same model, BI's support for the incorrect tree increased dramatically, while ML's did not. Finally, when data were simulated using the heterotachous model but analyzed using a standard homotachous model, support for the incorrect tree grew even stronger using BI but remained low using ML. These results indicate that 1) the empirical branch lengths alone are sufficient to cause bias in BI even when the underlying evolutionary model is simple, 2) this problem is exacerbated when the evolutionary process is complex, and 3) the stronger effect of model violation on BI further magnifies the bias.

### Bayesian Simulations

**Figure 4:**
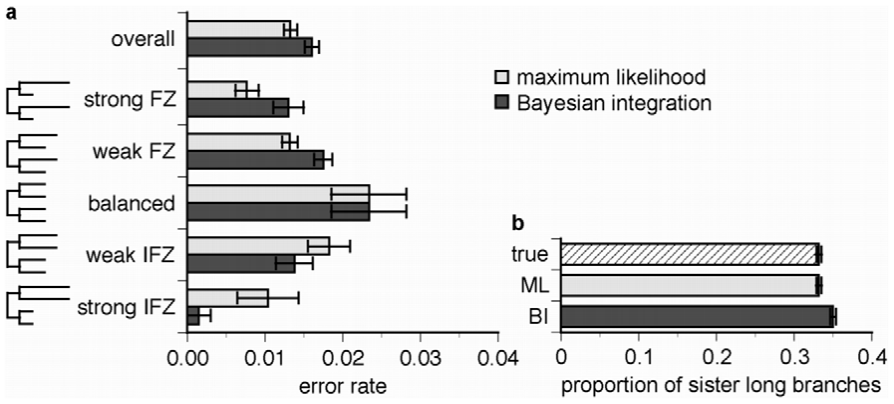
Bayesian integration is biased in “Bayesian simulation” when prior distributions are correctly specified. We simulated 5,000-nt sequences along randomly-selected four-taxon trees with branch lengths drawn from a uniform distribution on (0,1]. **a**, Datasets were divided into strong and weak Felsenstein zone (FZ) and inverse Felsenstein zone (IFZ) groups based on the pattern of branch lengths on the true tree and the difference between branch lengths (see Methods). The proportion of replicates in each category from which ML and BI recovered an incorrect phylogeny is shown. Bars indicate standard error. **b**, The proportion of datasets from which each method inferred the topology with the two longest terminal branches as sister taxa. The label “true” indicates the proportion of datasets for which the true tree has the two longest branches as sister taxa.

BI's long branch attraction bias has not been apparent in recent studies that used “Bayesian simulation” to generate sequence data on topologies and branch lengths drawn from probabilistic distributions rather than under specific conditions. These studies have found that when the prior distributions assumed for analysis match the true distributions, the mean posterior probability of a group of trees inferred using BI accurately reflects the fraction of those trees that are correct [29], [31]. To determine whether this approach to evaluating accuracy might mask an underlying bias under specific conditions, we conducted Bayesian simulations on four-taxon trees and partitioned the results according to the pattern of branch lengths on the true tree (Fig. 4). We found that when the lengths of non-sister branches are more similar to each other than to those of sister lineages (i.e., in the “Felsenstein zone”), BI recovered a false phylogeny significantly more often than ML, because of a specific bias in favor of the LBA tree. In contrast, when the lengths of sister branches were more similar to each other than to non-sister lineages (i.e., in the “inverse Felsenstein zone”), BI was more likely to recover the true tree than ML, because the LBA bias favors that tree. In this way, BI is similar to maximum parsimony, which outperforms ML in the inverse Felsenstein zone only because it is subject to a strong bias [24]. These results show that, even under the ideal conditions of Bayesian simulations in which the true prior distributions are used to analyze data, BI behaves like an estimator that systematically overestimates the value of a parameter under a specific set of conditions and underestimates it under the opposite conditions: the mean of all the estimates is accurate, but the estimates themselves are biased, a fact not apparent when only the mean of estimates is considered.

**Figure 5:**
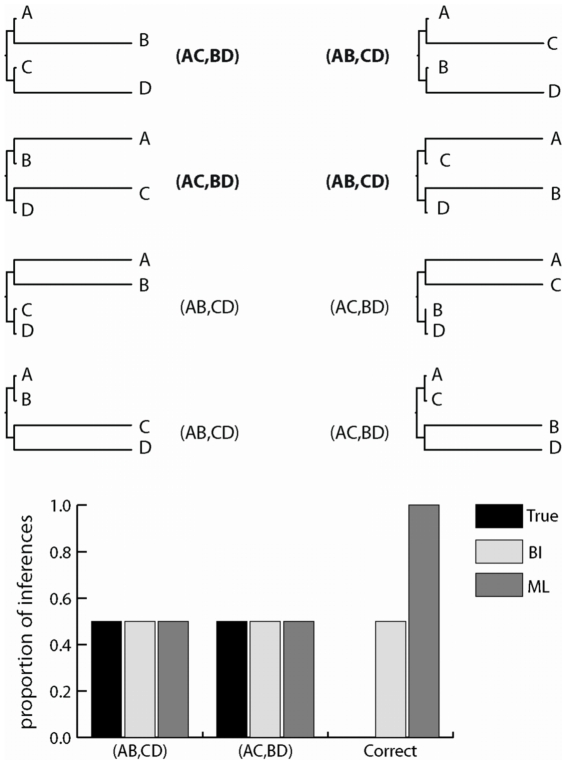
Bayesian integration is biased in a simplified Bayesian simulation. For each replicate, a topology/brach-length combination was chosen from a discrete set of sixteen, each with equal probability. There are two possible topologies (AB,CD) and (AC,BD); for each, there are four combinations of long (0.75 substitutions/site) and short (0.01) terminals, and two internal branch lengths (0.1 or 0.001, not shown) for each combination of terminal lengths. For each replicate, an ideal dataset with the expected state pattern frequencies was generated given the topology and branch lengths. When these data are analyzed using BI, with the true uniform distribution over the true set of topology/branch-length combinations used as a prior, the topology noted next to each tree is inferred as the maximum a posteriori phylogeny with support >0.99. Bold text indicates incorrect inferences; regular text, correct inferences. The chart shows the proportion of inferences from which each topology is recovered by BI and ML, along with the fraction of those inferences that are correct.

BI can therefore have a high error rate in Bayesian simulations, despite the correspondence between the fraction of inferences in which some topology *t* is inferred and the fraction of inferences in which *t* is true. This phenomenon occurs because the errors caused by BI's bias are equally distributed among possible topologies in a Bayesian simulation. A simplified example of Bayesian simulation illustrates this situation (Fig. 5). For each replicate, a phylogeny with branch lengths is chosen from a set of sixteen possibilities that have equal probability: on each of two four-taxon topologies (AB/CD or AC/BD), there are eight possible sets of branch lengths, half in the Felsenstein zone and half in the inverse Felsenstein zone. Sequence data with the ideal pattern frequencies are generated on that tree, which are then analyzed by ML or by BI using as a prior the true probability distribution of the sixteen possible phylogeny/branch length combinations. Under these conditions, ML infers the correct tree from all replicates, for an error rate of zero. In contrast, BI infers the correct tree only when the true tree is in the inverse-Felsenstein zone. For all replicates in the Felsenstein zone, BI incorrectly infers the AC/BD tree with very strong support when the AB/CD tree is true, and it infers AB/CD when AC/BD is true. BI's total error rate is therefore 50%. Because Felsenstein zone conditions are equally distributed across possible topologies, however, the frequency of errors in favor of AB/CD exactly compensates for the frequency of errors in favor of AC/BD, so BI accurately infers that the frequency of each topology is 50% over all replicates.

This example illustrates how integrating over branch lengths, even when the correct distribution is used as a prior, can result in strong bias and a very high rate of erroneous inferences. This problematic behavior is not apparent, however, when accuracy is measured only as the correspondence between the proportion of replicates in which some topology is true and the proportion of replicates from which it is inferred.

### Misinterpretation of Phylogenetic Signal

**Figure 6:**
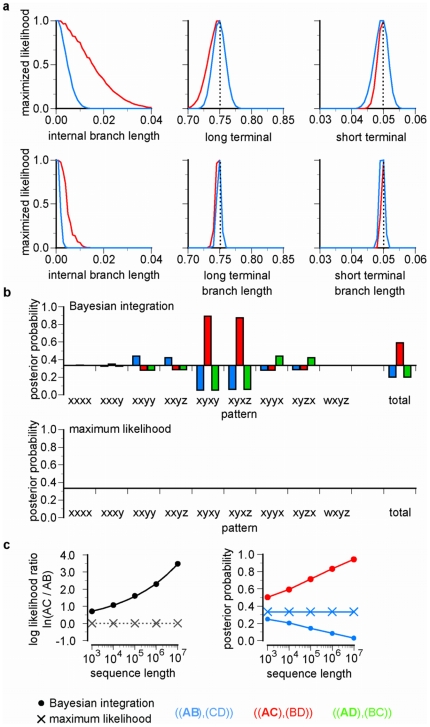
Integrating over branch length uncertainty causes misinterpretation of convergence as phylogenetic signal. We estimated the likelihood surface over branch lengths for datasets with expected character state pattern frequencies on a four-taxon star tree with long branches (0.75 substitutions/site) to termini A and C and short branches (0.05) to termini B and D. **a**, For each branch length, the likelihood is plotted for each of the three resolved trees, with the other lengths fixed at their ML values. Vertical dotted lines indicate the true branch lengths used to generate data. Likelihood functions are shown for expected datasets of N = 10,000 (top) and 100,000 (bottom). In both cases, the area under the curve for the long-branch attraction topology (red) exceeds that for the other topologies (blue and green, which are identical). **b**, The partial posterior probability of each resolved topology is shown for each character state pattern when branch lengths are integrated over (top) or fixed at their estimated values (bottom). Character state patterns are indicated using variables representing nucleotides of the same type: for example, pattern *xyxy* stands for the realizations ACAC, AGAG, ATAT, CACA,… TGTG. Results are shown for the expected 10,000-nt dataset. **c**, The log likelihood ratio of the long branch attraction tree (AC) to the AB tree is shown (left panel) for expected data of increasing sequence length generated on the star phylogeny. Right panel, corresponding posterior probability of each tree topology.

Correct phylogenetic inference using likelihood-based methods requires accurate branch length estimates. To understand how and why incorporating branch length uncertainty causes bias, we characterized the likelihood surface across branch lengths for sequences with the expected pattern frequencies generated on the star tree in Fig. 1a. As Fig. 6a shows, integrating over internal branch lengths causes LBA. At the true internal branch length of zero, the three possible trees have equal likelihood, but incorporating longer internal branch lengths causes the integrated likelihood of the LBA tree to dramatically exceed those of the other topologies. Integrating over the long and short terminal branches, in contrast, does not favor the LBA tree.

These results suggest that integrating over too-long internal branch lengths causes the convergent state patterns that occur on the long terminals to be misinterpreted as phylogenetic signal. To test this hypothesis, we calculated the partial posterior probability for each tree contributed by each character state pattern (Fig. 6b). When branch lengths were fixed at their ML values, none of the patterns produced strong support for any tree. When branch lengths were integrated over, however, patterns such as *xyxy* or *xyxz* provided strong support for the topology that clusters taxa with identical states. This result occurs because, when the internal branch is longer than its true value, the probability of such patterns is greater on the LBA topology than on the others. The net effect of incorporating incorrect internal branch lengths by Bayesian integration is therefore to misinterpret convergent patterns that arise on long branches as due to common descent. Although ML's estimates of branch lengths may deviate slightly from the true branch lengths due to stochastic variation in finite data, these deviations are apparently small and do not cause substantial topological bias.

### Increasing Bias with Larger Datasets

Our observation (Fig. 1) that the biasing effect of integrating over branch lengths grows worse with increasing sequence length may seem surprising, because the likelihood function over branch lengths for each topology becomes more peaked as sequence length grows (see Fig. 6a). The relative support for one tree over another, however, is determined by the ratio of the integrated likelihoods for the two topologies, modified by the priors. As the quantity of data grows, the likelihood function becomes more peaked for all topologies, and the ratio of the integrated likelihoods (and therefore of the posterior probabilities) in fact grows more extreme. For a dataset of length 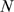 containing each state pattern *x* at the expected frequency 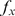, the integrated log-likelihood of any topology *j* is 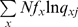 = 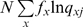, where 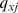 is the probability of pattern 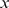 on topology 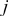 integrated over branch lengths *b*, or 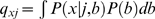. The log-likelihood ratio of any two trees 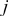 and 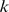 is 
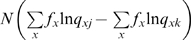
. The terms inside the parentheses—each state pattern's frequency in the expected data times its log-likelihood given each combination of topology and branch lengths—do not change with sequence length. The likelihood ratio must therefore scale exponentially with 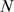, and the posterior probability of the favored tree must also increase towards the limit 1.0 as sequence length grows. If the expected state pattern frequencies support an incorrect tree at small sequence lengths—as our simulation experiments and numerical analyses indicate they do for Bayesian analysis of trees in the Felsenstein zone—then this support will grow more extreme, not less, as the quantity of data grows.

To corroborate this analysis, we numerically estimated the likelihood surfaces of expected datasets of increasing length, each composed of character state patterns at their expected frequencies given the Felsenstein-zone star tree in Fig. 1a. When branch lengths are integrated over, the likelihood ratio in favor of the LBA tree increases as sequence length grows (Fig. 6c), and the posterior probability of the LBA tree rises accordingly. Maximum likelihood estimation of branch lengths, in contrast, does not erroneously support one tree over the others.

## Discussion

Our results suggest that several of the proposed advantages of BI over ML for choosing among hypotheses of phylogeny are false. We found that integrating over branch length uncertainty does not improve accuracy but rather causes topological bias and reduced efficiency. In contrast, ML was not biased on finite data, and the asymptotically unbiased nature of ML is already well established [9], [15]–[17]. We found that BI's bias grows more severe as the amount of data increases, a particular concern in the age of phylogenomics [48]. Although this bias is relatively weak when the evolutionary model is simple, it becomes strong when more complex heterogeneous models are used, undermining the view that BI is preferable to ML for implementing mixed and partitioned models [3], [10], [12], [14]. Integrating over uncertainty also makes BI more susceptible than ML to errors caused by the inevitable use of inaccurate models of molecular evolution. ML's advantage over BI is apparent under empirical conditions and when small datasets are analyzed using complex models. In practice, ML and BI are likely to produce similar—and accurate—results most of the time, but when they differ, BI's inferences of topology are more likely to be due to bias than ML's. Our results indicate that BI will suffer from bias whenever the true tree contains non-sister long branches, a common occurrence in phylogenetics [46], [49], [50].

Unlike recent examinations of Bayesian phylogenetic approaches [30], [31], [51], which highlight potential problems with current MCMC implementations or prior distributions, our results point to problematic behavior that is intrinsic to Bayesian phylogenetics. The biases we observed cannot be alleviated with more sophisticated MCMC algorithms or complex prior distributions. BI is biased in favor of the LBA tree even when the correct prior assumptions are used in “Bayesian simulation.” A recent theoretical study showed that under the limiting distribution for a star tree with two long branches under the Jukes-Cantor model, the posterior probability of the LBA tree is higher than that of any other tree, including the star tree itself, irrespective of the specific prior distribution used for branch lengths [41]. Our experiments reveal the cause of this bias, show that BI-based phylogenetic inference is less accurate than ML, and establish that BI's bias affects accuracy on resolved trees, grows more severe with complex models, causes recovery of an incorrect phylogeny under empirical conditions, and makes BI more susceptible to error induced by model violation than ML.

Our results suggest that BI using a Bayes decision rule to choose among phylogenetic hypotheses may be statistically inconsistent. The proof of ML's consistency is based on the fact that when the evolutionary model is correct and identifiable, pattern frequencies in the data approach expectation as sequence length approaches the limit; under such conditions, maximum likelihood estimates of branch lengths converge on their true values, and the true topology with the true branch lengths always has higher likelihood than any other topology with any branch lengths [9]. This proof cannot apply to BI, because likelihoods are integrated over a distribution of branch lengths, the vast majority of which are wrong. A formal demonstration that BI is inconsistent in the Felsenstein zone is beyond the scope of this paper. However, our numerical and mathematical analyses show that when ideal data with the same properties as infinitely long sequences are analyzed, BI recovers the wrong phylogeny, and support for this erroneous topology increases as sequence length grows. Our simulations also show that BI recovers an incorrect phylogeny with increasing support as the amount of data increases, as expected for an inconsistent method but not a consistent one. Together with a previous analysis of the limiting distribution of posterior probabilities for data generated on a star tree in the Felsenstein zone [41], our findings provide strong, albeit circumstantial, evidence that BI is statistically inconsistent.

Although our results suggest problems with using BI for inferring phylogenies in practice, they do not contradict the core rationale for Bayesian inference. Bayes' Theorem defines posterior probability as the probability that a hypothesis is true given the model and the priors. If the priors on nuisance parameters match the true values of those parameters, Bayesian choice of topology will be unbiased and optimal, and the posterior probability of a topology will correspond to the probability that the tree is true given the data [32]. When the priors on nuisance parameters are incorrect, the posterior probability no longer holds this objective meaning, and the probability of the true topology is no longer guaranteed to exceed that of any other topology. Nevertheless, the posterior probability retains its purely subjective, conditional meaning as the degree of belief a rational agent will have in the hypothesis given whatever priors have been used. Ideally, prior distributions would accurately represent beliefs about the likely values of branch lengths and other model parameters before the data are analyzed, giving posterior probabilities a subjective meaning that is more than arbitrary. In reality, however, there is seldom reliable a priori information about model parameters, so it is typical for diffuse distributions to be used. Our results indicate that BI as currently practiced with such priors produces strongly biased inferences of topology under certain conditions. Alternative prior distributions [30], [31] are not effective at eliminating this bias.

BI would be unbiased if and only if the true length of every branch on any tree were known in advance and could be assigned as a prior for that branch with probability 1.0 [32]. That is, integrating over uncertainty would not cause bias if there were no uncertainty to integrate over. In reality, this situation can never be realized; if it could be, phylogenetic analysis would be unnecessary. Even if we could somehow know the “true distribution” of branch lengths on the universe of all phylogenies—that is, if the idealized circumstances of Bayesian simulations could be made real—our results show that BI would be systematically biased whenever some branches are arranged in Felsenstein-zone patterns, leading to an increased rate of topological error overall. This problematic behavior occurs because BI's accuracy depends upon explicit assumptions about the distributions of branch lengths, and these assumptions are wrong for most specific datasets even if they are correct on average. ML is not subject to this bias, because it makes no assumptions about the values of model parameters a priori. By inferring branch lengths from the data with reasonable accuracy, ML approximates the ideal situation in which branch lengths are known in advance. For biologists seeking to accurately infer historical relationships, these findings suggest that ML should generally lead to lower rates of error and systematic bias compared to BI.

Another proposed advantage of BI is that posterior probabilities provide a naturally meaningful measure of confidence in phylogenetic hypotheses. Integrating over uncertainty about branch lengths, however, causes inferred posterior probabilities to deviate radically from the probability that a tree or clade is true and, under some conditions, to favor an incorrect tree. In contrast, an empirical Bayesian approach using ML branch-length estimates yields posterior probabilities that better match this intuitive expectation [31], [32], [34]. The results presented here and in our prior work [32] suggest that empirical Bayesian approaches may provide a reliable alternative for calculating posterior probabilities of phylogenetic hypotheses, but further research is warranted.

A major practical advantage of BI has been the speed of MCMC-based analysis. Dramatic improvements in ML optimization methods now allow analysis of very large datasets [52], [53], although these methods calculate support measures other than posterior probabilities. Our empirical Bayes/ML software does calculate posterior probabilities, but it is time-consuming in its current implementation: analysis of the simulated eukaryote data took an average of 25 hours/dataset but only 2 hours using BI. Future improvements may reduce the computational demands of this approach.

There are solid philosophical arguments in favor of both Bayesian and likelihood-based approaches to scientific inference [54], [55]. Phylogenetics, with its hierarchical branching structure, presents a peculiar realm of statistical analysis, where mistaking noise for signal and integrating over uncertainty about nuisance parameters can lead to systematically biased inferences. Philosophical considerations notwithstanding, our results suggest that one of the key conceptual advantages of BI over ML makes it less reliable in practice.

## Methods

### Phylogenetic Analyses

*Bayesian Inference (BI)* phylogenies were inferred using MrBayes 3.1.2 [43]. Priors were set at default values except for branch lengths, which were assumed to be uniformly distributed between zero and 10 substitutions/site. Additional branch length priors were also explored, including uniform distributions with various upper bounds, exponential priors with various means, and novel priors that assign independent distributions to terminal and internal branch lengths. For each analysis, four incrementally heated chains were used per run, with samples taken from the cold chain every 100 generations. The first 100 samples were excluded as burn-in, and analyses were terminated when the standard deviation in clade posterior probabilities between two independent runs dropped below 0.01. The correct evolutionary models of relative substitution probabilities, equilibrium state frequencies, and among-site rate variation were used unless otherwise noted.

*Maximum Likeihood (ML)* phylogenies were inferred using novel “empirical Bayes” MCMC software (available for download at http://phylo.uoregon.edu/software/eb). For each replicate, a single cold chain was started from the neighbor joining topology and sampled every 10 generations—without burnin—until the average posterior probability of each tree topology remained constant (within a margin of 0.01) for 10 samples. At each generation, a new tree topology was proposed using a subtree-purining-regrafting (SPR) operation, in which a randomly-selected subtree is removed from the current topology and then re-attached at a randomly-selected position. Branch lengths and other parameters on this proposed topology were optimized using Phyml [56]. Proposed topologies were accepted or rejected based on the Metropolis criterion, assuming prior probability 1.0 on the maximum likelihood value for each nuisance parameter and equal prior probability on each resolved tree topology. The Hastings ratio for this proposal mechanism is 1.0, as each possible SPR pruning and regrafting position has an equal probability of being selected, and proposed branch lengths are not conditional on the current lengths. As with BI, the correct evolutionary model was used for each analysis, unless otherwise indicated.

*Partitioned Analyses* were conduced using MrBayes for BI and a pre-release version of RAxML [53] for ML. In the case of simulated data, sites were correctly partitioned, with branch lengths treated separately for sites in each partition. Other model parameters were correctly assumed to be equivalent across all sites. Priors and other analysis parameters were the same as for homogeneous BI and ML analyses.

*The Mixed Branch Length Model* calculates a weighted sum of likelihoods for multiple independent sets of branch lengths on the tree; the likelihood of tree *t* given data *X* = (*x* 1, *x* 2, …, *x* m) and branch length sets *b* = (*b* 1, *b* 2, …, *b n*) is given by
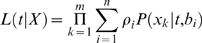
where each 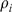 is estimated from the data, and 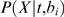 is the probability of the data given branch lengths 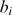. Bayesian mixed model analysis was conducted using a pre-release version of BayesPhylogenies [57] for BI. Analyses were conducted as above, with the exception that heated chains and multiple runs were not used. Instead, a single cold chain was run for 100,000 generations. Default priors were used for mixture proportions. ML mixed model analyses were conducted as above, with branch lengths, mixture proportions and other parameters optimized using custom software implementing a simulated annealing algorithm [47]. The annealing schedule used a geometric descent of 500 temperatures from a high of 1.0 to a low of 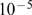. At each temperature, 500 parameter changes were proposed, with acceptance based on the Metropolis criterion. The empirical Bayes posterior probability of each of the three trees was then calculated numerically from these point likelihoods.

### Four-Taxon Simulations

We simulated a variety of nucleotide and amino acid data sets along a four-taxon ((AB),(CD)) phylogeny with long terminal branches (0.75 substitutions/site) leading to taxa B and D and short terminals (0.05) leading to A and C. The internal branch length varied from 0.0 to 0.05. Sequence length varied from 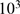 to 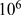. Evolutionary models used to simulate nucleotide data included the JC69 model, JC69 with 25% invariant sites, JC69 with gamma-distributed among-site rate variation (8 discrete category approximation, shape parameter 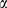 = 0.5), K80 (transition/transversion ratio = 10.0), F81 (80% G+C), and HKY85 (transition/transversion = 10.0, G+C = 80%). Amino acid sequences were simulated using the empirical JTT model. For each set of conditions, 500 replicate sequence alignments were generated.

We also simulated sequences along the ((AB),(CD)) phylogeny with heterogeneous evolutionary pressures across either sites or lineages. Across-site heterogeneity included 1) conditions in which 1/2 of sites had 45% G+C, while the other 1/2 had 5% G+C, and 2) conditions in which a proportion *p* of sites had long terminal branches (0.75) leading to taxa B and D and short terminal branches (0.05) leading to A and C, while the remaining sites had long branches A and C and short branches B and D. We varied the mixture proportion *p* from 0.0 (no heterogeneity) to 0.5 (maximal heterogeneity). For across-lineage heterogeneity, we simulated nucleotide sequences with 30% G+C content; in non-sister lineages B and D, G+C content was increased by a variable amount, from no G+C increase up to a maximal increase of 70% (producing sequences with 100% G+C).

### Eukaryote Elongation Factor 1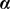 (EF1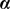) Analyses

We analyzed the Micro* data set of ref. [45] using homotachous ML and BI, as described above. Mixed branch length model analyses were conducted in both ML and BI frameworks using previously described software [47]. In the case of ML, simulated annealing was used to optimize the tree topology and all model parameters. The annealing schedule used a geometric descent of 1000 temperatures starting from 1.0 and ending at 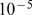. At each temperature, 1000 parameter changes were attempted, with acceptance based on the Metropolis criterion. Topology proposals included TBR, SPR, and NNI. The best-fit number of branch length classes (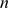) was estimated using the Akaike Information Criterion (AIC) [58].

The same parameter and tree proposal mechanisms were used for BI analysis. Priors were uniform over resolved topologies and uniform on (0,10] for branch lengths. The MrBayes default prior—U[0.05,200]—was used for the shape parameter of the gamma distribution. A flat Dirichlet prior was assumed for the mixture proportions (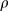).

ln-Bayes Factors (lnBFs) of the correct Microsporidia+Fungi (MF) tree to the artifactual Microsporidia+Archaebacteria (MA) tree were calculated assuming equal priors on both tree topologies as 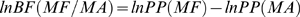, where 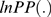 is the log of the posterior probability of either the MF or MA tree, calculated using either ML or BI. In the case of ML analysis, the ln-Bayes Factor (assuming equal priors over topologies) is equivalent to the ln-likelihood ratio: 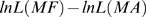.

Partitioned analyses were conducted by partitioning sites based on mixed model analysis. Using the maximum likelihood topology inferred under the model selected by AIC, we calculated the posterior probability that each site evolved according to each set of inferred branch lengths. The posterior probability of branch length set 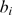 given site 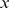 was calculated by multiplying the proportion of sites expected to evolve under branch length set 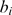 (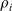) by the likelihood obtained for that branch length set (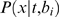) and dividing by the total likelihood summed over all branch length sets:
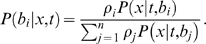



We used a posterior probability cutoff of 0.95 to classify sites into categories: a site 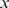 was assigned to a particular class 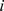 if the posterior probability of that class (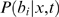) was greater than 0.95. Sites not classified with 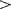
0.95 posterior probability were excluded from the analysis. Partitioned analyses were conduced using MrBayes for BI and a pre-release version of RAxML for ML, as described above. Branch lengths were treated separately for sites in each partition. Other model parameters were assumed to be equivalent across all sites. Priors and other analysis parameters were the same as for homotachous BI and ML analyses.

### Bayesian Simulations

We performed Bayesian simulations [29] of 5,000-nt datasets along a four-taxon phylogeny using the JC69 model. For each of 20,000 replicates, the topology was selected at random, and branch lengths were randomly drawn from a uniform distribution on (0,1]. For each dataset, we calculated the strength of branch length heterogeneity as 
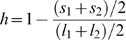
 where 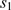 and 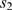 are the two shorter terminal branch lengths on the topology, and 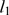 and 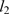 are the two longer terminal branches. We used the type and degree of branch length heterogeneity to divide data sets into five classes. 1) Strong Felsenstein zone (FZ) replicates had long terminal branches in non-sister lineages with branch length heterogeneity 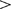
0.75. 2) Weak FZ replicates had non-sister long branches with branch length heterogeneity between 0.75 and 0.25. 3) Balanced replicates had branch length heterogeneity 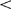
0.25. 4) Weak inverse Felsenstein zone (IFZ) data had sister long terminal branch lengths with branch length heterogeneity between 0.25 and 0.75, and 5) strong IFZ data had sister long branches with heterogeneity 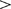
0.75.

For the simplified example of Bayesian simulation, the generating conditions included two topologies—(AB,CD) and (AC,BD). On each topology, there were eight possible branch length sets, comprised of two possible internal branch lengths (0.0001 or 0.1) and four possible sets of terminal branch lengths, each of which included two long (0.75) and two short (0.01) branches: A and B long, C and D long, A and C long, or B and D long. For each generating condition, an ideal pseudo-dataset—in which the frequency of each state pattern matches expectation given the JC69 model—was prepared by using Phyml to calculate the probability of each state pattern. For data generated under each condition, the exact likelihood of all 16 possible conditions, assuming the correct evolutionary model, was calculated as follows. The pattern-specific likelihood 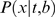) of each possible combination of topology *t* and branch lengths *b* given a single site with each state pattern *x* and the true model was calculated using Phyml. The likelihood at all sites with pattern *x* in a dataset of size *N* was then calculated as
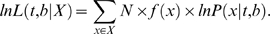
The total log-likelihood given a dataset of length 10,000 sites was calculated as the sum of the pattern-specific log-likelihoods over all patterns. From these likelihoods, the ML/empirical Bayes posterior probability of each topology was calculated using a point prior that places prior density 1.0 on the ML branch lengths for that topology, with priors for the two topologies matching the true generating conditions. The BI posterior probability of each topology was calculated by integrating over all eight branch length sets for that topology, using a prior distribution in which the probability of each topology and each branch length set on that topology matched the true generating conditions. Performance was evaluated over an ideal set of Bayesian simulations in which each generating condition appeared at its expected frequency.

### Likelihood Surface Estimation

We generated datasets of 1000 to 10 million nucleotides having the expected state pattern frequencies, given a four-taxon star tree with two long (0.75) and two short (0.05) branch lengths. We used Phyml and the JC69 model to calculate the expected frequency of each possible character state pattern (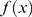) as described above. We characterized the likelihood surface of each possible resolved tree using numerical integration over branch lengths. The probability of each pattern and the total log-likelihood over all patterns was calculated for each combination of branch lengths on each possible four-taxon topology was calculated as described above for the simplified Bayesian simulation. For each topology, the likelihood surface was sampled using a branch length interval of 0.001 across a range of values giving significant likelihood. The internal branch was sampled between 0.0 and 0.04; long terminal branch lengths were sampled between 0.7 and 0.85, and short terminals were sampled between 0.03 and 0.06, inclusive. To reduce the computational burden of this experiment, branches with equivalent simulated lengths were assumed to be equal, reducing the dimensionality of the likelihood space. Integrating over larger intervals and all four terminal lengths independently had negligible effect on support for different topologies (data not shown). Likelihood values were arbitrarily scaled so that the maximum likelihood across all topologies and branch length values was 1.0.

## Supporting Information

Table S1 Bayesian integration (BI) requires more phylogenetic signal to recover the correct tree than maximum likelihood (ML). For each panel in Figure 1, we calculate the BL95-the internal branch length at which the correct phylogeny is recovered from 95% of replicates-for BI and ML using logistic regression: 1/{1 + e^∧^[(x−c)s]}, where x is the internal branch length; c is the internal length at which 50% accuracy is achieved, and s is the slope of the curve.
(0.02 MB PDF)

Figure S1 Maximum likelihood and Bayesian MCMC samplers differ in how they deal with nuisance parameters. **a,** In traditional Bayesian MCMC, proposals are made by altering the tree topology, branch lengths and/or other model parameters. The likelihood of the proposed state is compared to that of the current state and either accepted or rejected. The algorithm proceeds by iteration, and samples of the current state are taken at fixed intervals. The proportion with which a given topology is sampled provides an estimate of the tree's posterior probability. **b,** In maximum likelihood MCMC, only topology changes are proposed. Model parameters (including branch lengths) are then optimized on the proposed topology using maximum likelihood before comparing the proposed tree to the current tree and either accepting or rejecting the proposal.
(0.02 MB PDF)

Figure S2 Various evolutionary models produce long branch attraction bias when Bayesian integration (BI) is used; maximum likelihood (ML) is unbiased. The proportion of 500 replicates from which each possible resolved tree was recovered and mean posterior probability of each tree is plotted for BI and ML. Bars indicate standard error. Different evolutionary models were used to simulate data of 5,000 and 50,000 nucleotides on a star tree with two long (0.75 substitutions/site) and two short (0.05) terminal branches. Analyses were conducted using the true model in each case. The proportion of invariant sites for JC69+I was 0.25. The shape parameter (alpha) for JC69+G8 was 0.5. The transition/transversion ratio for K80 and HKY85 was 10.0. The G+C content for F81 and HKY85 was 80%.
(0.03 MB PDF)

Figure S3 Bayesian integration (BI) is biased when protein data are analyzed; maximum likelihood (ML) is unbiased. The proportion of 500 replicates from which each possible tree was recovered and mean posterior probability of each tree are plotted; bars indicate standard error. Sequence data of 5,000 and 50,000 amino acids were simulated on an unresolved star tree with two long (0.75 substitutions/site) and two short (0.05) terminal branches using the JTT model. Analyses were conducted using the true model.
(0.02 MB PDF)

Figure S4 Bayesian integration is biased when various branch length prior distributions are used. Exponential priors with mean values from 10^∧^-5 to 10.0 substitutions/site (left) and uniform priors with lower bound 0.0 and upper bounds from 1.0 to 100 were used on branch lengths. The proportion of 500 replicates from which each tree was recovered and mean posterior probability are plotted, with bars indicating standard error. Data were simulated using the JC69 model; the topology was unresolved, with two long terminal branch lengths (0.75 substitutions/site) and two short terminals (0.05). The true model was used to analyze data.
(0.03 MB PDF)

Figure S5 Non-standard prior distributions do not alleviate long branch attraction when Bayesian integration is used. Data were simulated using the JC69 model and an unresolved topology with two long (0.75 substitutions/site) and two short (0.05) terminal branch lengths. The true evolutionary model was used to analyze data. The proportion of replicates from which each possible resolved tree was recovered and mean posterior probability for each tree are shown; bars indicate standard error. **a,** Analyses were conducted using different prior distributions for internal and terminal branch lengths (ref. 1). The prior on the internal branch length was exponential with mean 10^∧^-5; the exponential prior on terminal lengths had mean 0.1. **b,** We altered the branch length proposal mechanism of MrBayes v3.1.2 to allow proposals of zero-length branches on each topology. Data were analyzed using a branch length prior uniform on [0,10]. c, Analyses were conducted using a Bayesian method that explicitly samples unresolved trees (ref. 2). Equal prior probability (0.25) was placed on the three possible resolved trees and the unresolved star tree. To estimate topological bias, recovery of the star tree as the best-supported topology was scored as 1/3 recovery of each resolved phylogeny, and the posterior probability for the star tree was equally distributed among the resolved trees for each replicate.
(0.03 MB PDF)
