# Correction: Long-Branch Attraction Bias and Inconsistency in Bayesian Phylogenetics

**DOI:** 10.1371/annotation/93635f70-47d7-4876-84f5-dd568434c9ae

**Published:** 2010-03-01

**Authors:** Bryan Kolaczkowski, Joseph W. Thornton

The Section titled "Increasing Bias with Larger Datasets" has been revised. The corrected text should read: The expression we used on page 7891 for the log-likelihood ratio of two topologies j and k integrated over branch lengths is incorrect. Please view the correct expression here: 

**Figure:**
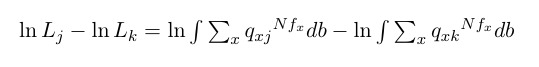


where q_xj is the probability of state pattern x given branch lengths b on tree j, fx is the frequency of pattern x in the dataset, and N is the number of sites in each sequence. Our expression erroneously integrates log-likelihoods over branch lengths; the correct expression integrates likelihoods before taking their logarithms. As a result, our conclusion in the accompanying text that the log-likelihood ratio integrated over branch lengths given expected data scales linearly with N is incorrect. The numerical integrations depicted in Figures 6c and 6d also used the incorrect expression to calculate log-likelihoods.

We have recalculated the integrated log-likelihoods using the correct expression. The revised results for Figure 6 are available here:

**Figure:**
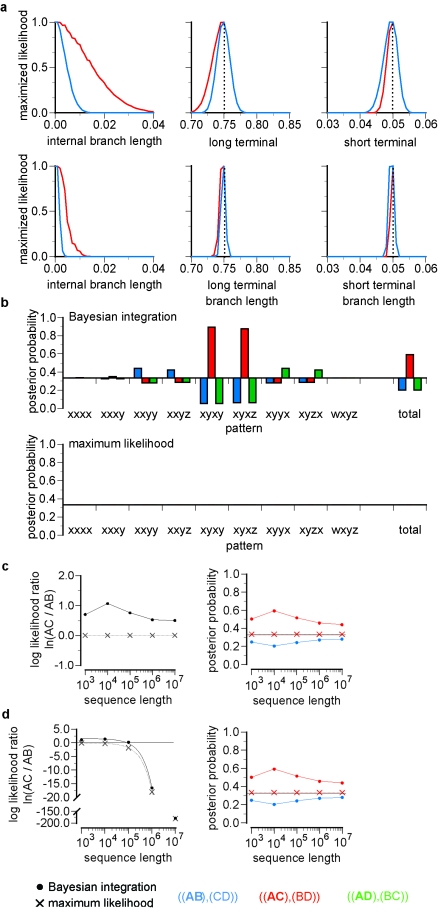


The corrected results indicate that when the true is unresolved, BI is biased given expected data in the Felsenstein zone, and this bias neither increases nor declines rapidly as sequence lengths increase (Fig. 6c*); this result is consistent with our findings using BMCMC (Fig. 1). We have conducted a similar analysis for rooted trees: in this case, support for the true tree increases as the amount of sequence data grows, eventually outweighing the bias observed at shorter sequence lengths. For expected data generated on a resolved tree with strong Felsenstein-zone branch lengths (Fig. 6d*), BI incorrectly infers the LBA tree as the maximum a posteriori tree at sequence lengths below Note: 0000100,000 sites; above this threshold, the true is inferred, with increasing support as sequence length grows. On both resolved and unresolved trees, BI's bias makes it less efficient than ML, recovering the true tree less reliably and with lower support at any given sequence length.

This error does not affect other results in our paper, which were obtained using MCMC. We conclude that BI is biased but unlikely to be asymptotically inconsistent, so long as the true tree is resolved and the true model is used; whether BI is consistent when the true tree is unresolved remains an open question. The amount of sequence data required for BI to infer the true tree under strong Felsenstein-zone conditions can be large even when the model is simple, although not larger than found in phylogenomic-scale datasets. Fewer sites will be required with less extreme branch-length conditions, but the bias becomes considerably stronger when the true model is complex. Under Felsenstein-zone conditions with datasets of realistic size, BI appears to be less efficient than ML, which does not display BI's topoloical bias at finite sequence lengths.

We are grateful to Mike Steel for his help in discovering this error. 

