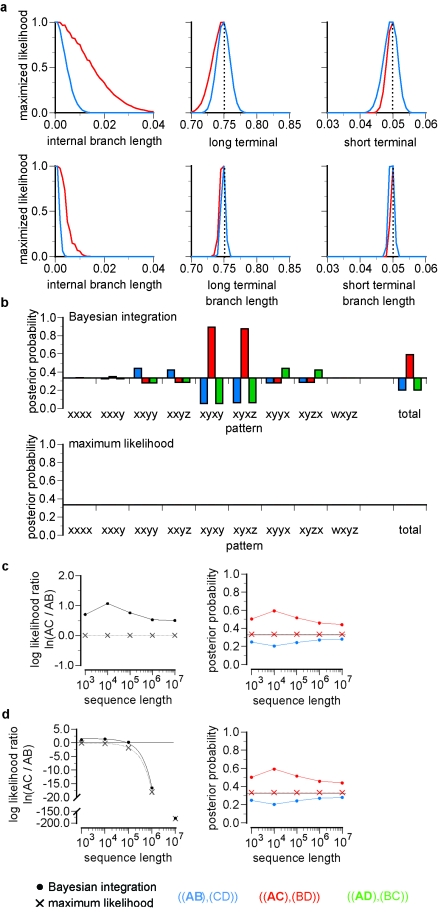# Correction: Long-Branch Attraction Bias and Inconsistency in Bayesian Phylogenetics

**DOI:** 10.1371/annotation/fb21ff39-0500-49c9-9ab3-21d924c6299d

**Published:** 2010-03-30

**Authors:** Bryan Kolaczkowski, Joseph W. Thornton

The results in the section titled "Increasing Bias with Larger Datasets" have changed, which affects the data in Figure 6. Please view the corrected Figure 6 here: 

**Figure:**