# Correction: Long-Branch Attraction Bias and Inconsistency in Bayesian Phylogenetics

**DOI:** 10.1371/annotation/1cd49dad-a033-4832-b2a9-610dc84e2662

**Published:** 2010-06-08

**Authors:** Bryan Kolaczkowski, Joseph W. Thornton

The legend for Figure 6 contained inaccuracies. The correct Figure 6 legend should read: Integrating over branch length uncertainty causes misinterpretation of convergence as phylogenetic signal. We estimated the likelihood surface over branch lengths for datasets with expected character state pattern frequencies on a four-taxon star tree with long branches (0.75 substitutions/site) to termini A and C and short branches (0.05) to termini B and D. a, For each branch length, the likelihood is plotted for each of the three resolved trees, with the other lengths fixed at their ML values. Vertical dotted lines indicate the true branch lengths used to generate data. Likelihood functions are shown for expected datasets of N = 10,000 (top) and 100,000 (bottom). In both cases, the area under the curve for the long-branch attraction topology (red) exceeds that for the other topologies (blue and green, which are identical). b, The partial posterior probability of each resolved topology is shown for each character state pattern when branch lengths are integrated over (top) or fixed at their estimated values (bottom). Character state patterns are indicated using variables representing nucleotides of the same type: for example, pattern xyxy stands for the realizations ACAC, AGAG, ATAT, CACA,…TGTG. Results are shown for the expected 10,000-nt dataset. c, The log likelihood ratio of the long branch attraction tree (AC) to the AB tree is shown (left panel) for expected data of increasing sequence length generated on the star phylogeny. Right panel, corresponding posterior probability of each tree topology. Filled circles, BI; crosses, ML. d, Support for each topology is shown under similar conditions for a resolved true tree (internal branch length 0.001).

