# Supplementary material for: Long-Branch Attraction Bias and Inconsistency in Bayesian Phylogenetics
Source: PLoS One. 2009 Dec 9;4(12):e7891. doi: 10.1371/journal.pone.0007891 (PMC2785476; doi:10.1371/journal.pone.0007891)
Supplement: Table S1 — Bayesian integration (BI) requires more phylogenetic signal to recover the correct tree than maximum likelihood (ML). For each panel in Figure 1, we calculate the BL95-the internal branch length at which the correct phylogeny is recovered from 95% of replicates-for BI and ML using logistic regression: 1/{1 + e∧[(x−c)s]}, where x is the internal branch length; c is the internal length at which 50% accuracy is achieved, and s is the slope of the curve. (0.02 MB PDF) [file pone.0007891.s001.pdf]

Figure 1 Panel	BI	ML	ratio(BI/ML)
a	0.020	0.016	1.25
b	0.027	0.021	1.27
c	0.024	0.018	1.38
d	0.038	0.019	2.02

Table S1 Bayesian integration (BI) requires more phylogenetic signal to recover the correct tree than maximum likelihood (ML). For each panel in Figure 1, we calculate the BL_{95}—the internal branch length at which the correct phylogeny is recovered from 95% of replicates—for BI and ML using logistic regression: $1/(1+e^{(x-c)s})$, where x is the internal branch length; c is the internal length at which 50% accuracy is achieved, and s is the slope of the curve.
