# Supplementary material for: Long-Branch Attraction Bias and Inconsistency in Bayesian Phylogenetics
Source: PLoS One. 2009 Dec 9;4(12):e7891. doi: 10.1371/journal.pone.0007891 (PMC2785476; doi:10.1371/journal.pone.0007891)
Supplement: Figure S1 — Maximum likelihood and Bayesian MCMC samplers differ in how they deal with nuisance parameters. a, In traditional Bayesian MCMC, proposals are made by altering the tree topology, branch lengths and/or other model parameters. The likelihood of the proposed state is compared to that of the current state and either accepted or rejected. The algorithm proceeds by iteration, and samples of the current state are taken at fixed intervals. The proportion with which a given topology is sampled provides an estimate of the tree's posterior probability. b, In maximum likelihood MCMC, only topology changes are proposed. Model parameters (including branch lengths) are then optimized on the proposed topology using maximum likelihood before comparing the proposed tree to the current tree and either accepting or rejecting the proposal. (0.02 MB PDF) [file pone.0007891.s002.pdf]

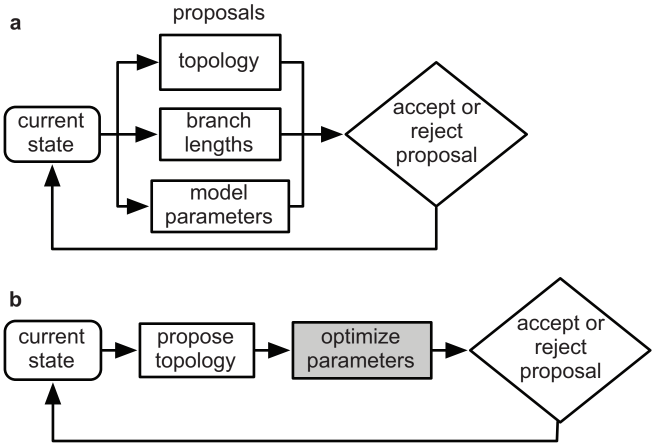


**Figure S1** Maximum likelihood and Bayesian MCMC samplers differ in how they deal with nuisance parameters. **a**, In traditional Bayesian MCMC, proposals are made by altering the tree topology, branch lengths and/or other model parameters. The likelihood of the proposed state is compared to that of the current state and either accepted or rejected. The algorithm proceeds by iteration, and samples of the current state are taken at fixed intervals. The proportion with which a given topology is sampled provides an estimate of the tree's posterior probability. **b**, In maximum likelihood MCMC, only topology changes are proposed. Model parameters (including branch lengths) are then optimized on the proposed topology using maximum likelihood before comparing the proposed tree to the current tree and either accepting or rejecting the proposal.
