# Supplementary material for: Long-Branch Attraction Bias and Inconsistency in Bayesian Phylogenetics
Source: PLoS One. 2009 Dec 9;4(12):e7891. doi: 10.1371/journal.pone.0007891 (PMC2785476; doi:10.1371/journal.pone.0007891)
Supplement: Figure S2 — Various evolutionary models produce long branch attraction bias when Bayesian integration (BI) is used; maximum likelihood (ML) is unbiased. The proportion of 500 replicates from which each possible resolved tree was recovered and mean posterior probability of each tree is plotted for BI and ML. Bars indicate standard error. Different evolutionary models were used to simulate data of 5,000 and 50,000 nucleotides on a star tree with two long (0.75 substitutions/site) and two short (0.05) terminal branches. Analyses were conducted using the true model in each case. The proportion of invariant sites for JC69+I was 0.25. The shape parameter (alpha) for JC69+G8 was 0.5. The transition/transversion ratio for K80 and HKY85 was 10.0. The G+C content for F81 and HKY85 was 80%. (0.03 MB PDF) [file pone.0007891.s003.pdf]

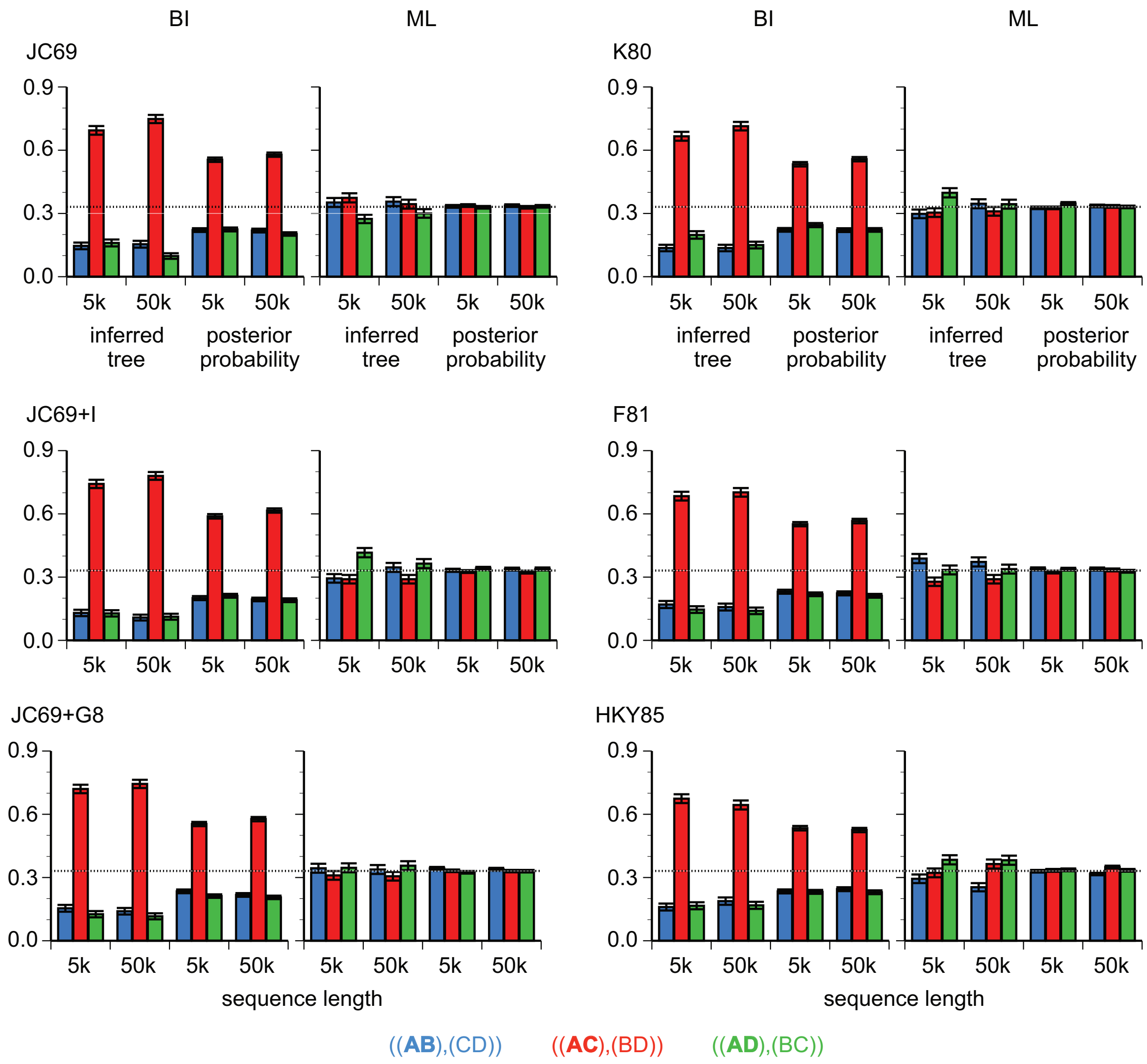

Figure S2 Various evolutionary models produce long branch attraction bias when Bayesian integration (BI) is used; maximum likelihood (ML) is unbiased. The proportion of 500 replicates from which each possible resolved tree was recovered and mean posterior probability of each tree is plotted for BI and ML. Bars indicate standard error. Different evolutionary models were used to simulate data of 5,000 and 50,000 nucleotides on a star tree with two long (0.75 substitutions/site) and two short (0.05) terminal branches. Analyses were conducted using the true model in each case. The proportion of invariant sites for JC69+I was 0.25. The shape parameter (α) for JC69+G8 was 0.5. The transition/transversion ratio for K80 and HKY85 was 10.0. The G+C content for F81 and HKY85 was 80%.
