# Supplementary material for: Long-Branch Attraction Bias and Inconsistency in Bayesian Phylogenetics
Source: PLoS One. 2009 Dec 9;4(12):e7891. doi: 10.1371/journal.pone.0007891 (PMC2785476; doi:10.1371/journal.pone.0007891)
Supplement: Figure S3 — Bayesian integration (BI) is biased when protein data are analyzed; maximum likelihood (ML) is unbiased. The proportion of 500 replicates from which each possible tree was recovered and mean posterior probability of each tree are plotted; bars indicate standard error. Sequence data of 5,000 and 50,000 amino acids were simulated on an unresolved star tree with two long (0.75 substitutions/site) and two short (0.05) terminal branches using the JTT model. Analyses were conducted using the true model. (0.02 MB PDF) [file pone.0007891.s004.pdf]

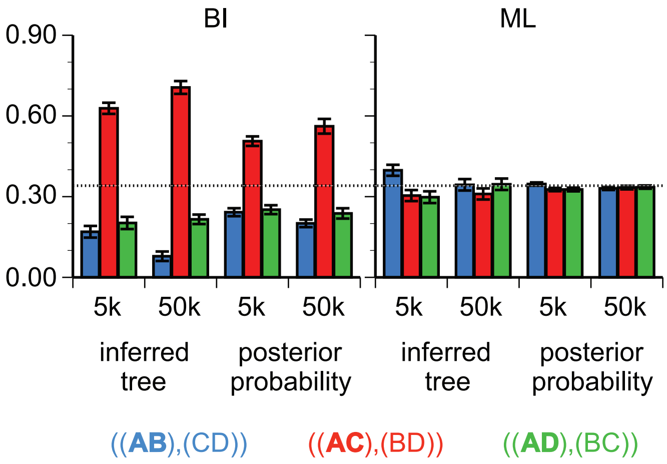

Figure S3 Bayesian integration (BI) is biased when protein data are analyzed; maximum likelihood (ML) is unbiased. The proportion of 500 replicates from which each possible tree was recovered and mean posterior probability of each tree are plotted; bars indicate standard error. Sequence data of 5,000 and 50,000 amino acids were simulated on an unresolved star tree with two long (0.75 substitutions/site) and two short (0.05) terminal branches using the JTT model. Analyses were conducted using the true model.
