# Supplementary material for: Long-Branch Attraction Bias and Inconsistency in Bayesian Phylogenetics
Source: PLoS One. 2009 Dec 9;4(12):e7891. doi: 10.1371/journal.pone.0007891 (PMC2785476; doi:10.1371/journal.pone.0007891)
Supplement: Figure S4 — Bayesian integration is biased when various branch length prior distributions are used. Exponential priors with mean values from 10∧-5 to 10.0 substitutions/site (left) and uniform priors with lower bound 0.0 and upper bounds from 1.0 to 100 were used on branch lengths. The proportion of 500 replicates from which each tree was recovered and mean posterior probability are plotted, with bars indicating standard error. Data were simulated using the JC69 model; the topology was unresolved, with two long terminal branch lengths (0.75 substitutions/site) and two short terminals (0.05). The true model was used to analyze data. (0.03 MB PDF) [file pone.0007891.s005.pdf]

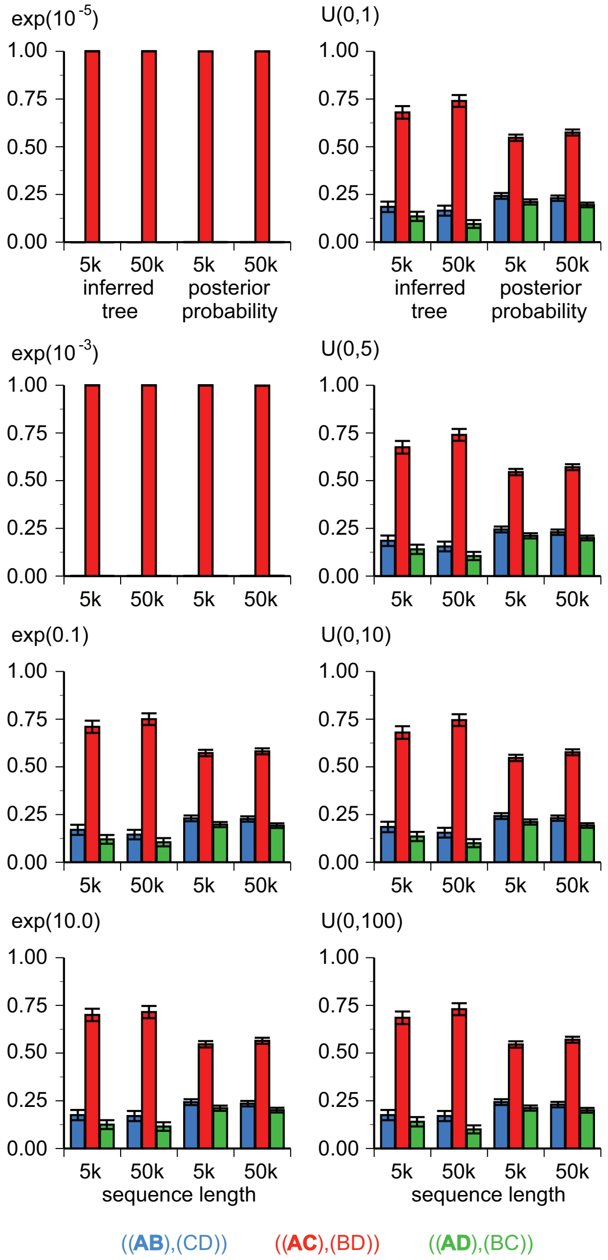

Figure S4 Bayesian integration is biased when various branch length prior distributions are used. Exponential priors with mean values from 10^{-5} to 10.0 substitutions/site (left) and uniform priors with lower bound 0.0 and upper bounds from 1.0 to 100 were used on branch lengths. The proportion of 500 replicates from which each tree was recovered and mean posterior probability are plotted, with bars indicating standard error. Data were simulated using the JC69 model; the topology was unresolved, with two long terminal branch lengths (0.75 substitutions/site) and two short terminals (0.05). The true model was used to analyze data.
