# Supplementary material for: Long-Branch Attraction Bias and Inconsistency in Bayesian Phylogenetics
Source: PLoS One. 2009 Dec 9;4(12):e7891. doi: 10.1371/journal.pone.0007891 (PMC2785476; doi:10.1371/journal.pone.0007891)
Supplement: Figure S5 — Non-standard prior distributions do not alleviate long branch attraction when Bayesian integration is used. Data were simulated using the JC69 model and an unresolved topology with two long (0.75 substitutions/site) and two short (0.05) terminal branch lengths. The true evolutionary model was used to analyze data. The proportion of replicates from which each possible resolved tree was recovered and mean posterior probability for each tree are shown; bars indicate standard error. a, Analyses were conducted using different prior distributions for internal and terminal branch lengths (ref. 1). The prior on the internal branch length was exponential with mean 10∧-5; the exponential prior on terminal lengths had mean 0.1. b, We altered the branch length proposal mechanism of MrBayes v3.1.2 to allow proposals of zero-length branches on each topology. Data were analyzed using a branch length prior uniform on [0,10]. c, Analyses were conducted using a Bayesian method that explicitly samples unresolved trees (ref. 2). Equal prior probability (0.25) was placed on the three possible resolved trees and the unresolved star tree. To estimate topological bias, recovery of the star tree as the best-supported topology was scored as 1/3 recovery of each resolved phylogeny, and the posterior probability for the star tree was equally distributed among the resolved trees for each replicate. (0.03 MB PDF) [file pone.0007891.s006.pdf]

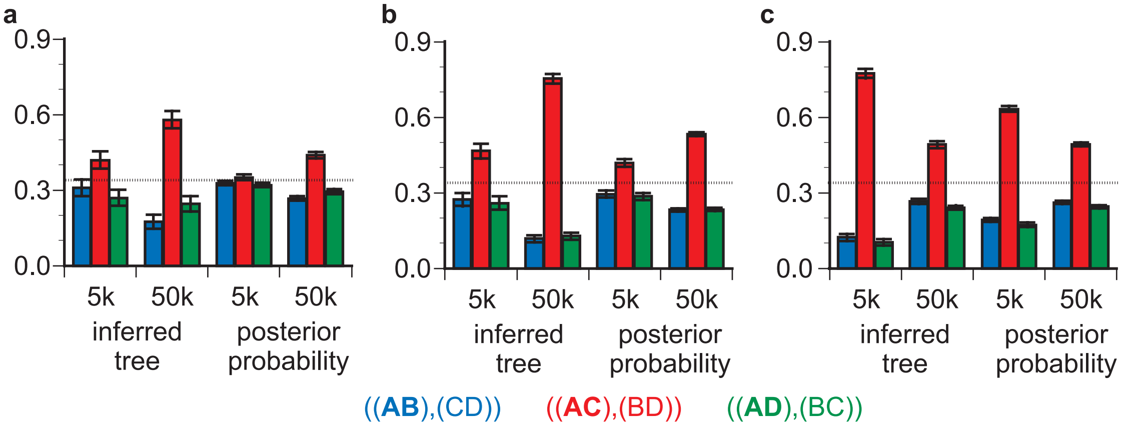

Figure S5 Non-standard prior distributions do not alleviate long branch attraction when Bayesian integration is used. Data were simulated using the JC69 model and an unresolved topology with two long (0.75 substitutions/site) and two short (0.05) terminal branch lengths. The true evolutionary model was used to analyze data. The proportion of replicates from which each possible resolved tree was recovered and mean posterior probability for each tree are shown; bars indicate standard error. **a**, Analyses were conducted using different prior distributions for internal and terminal branch lengths[1]. The prior on the internal branch length was exponential with mean 10^{-5}; the exponential prior on terminal lengths had mean 0.1. **b**, We altered the branch length proposal mechanism of MrBayes v3.1.2 to allow proposals of zero-length branches on each topology. Data were analyzed using a branch length prior uniform on [0,10]. **c**, Analyses were conducted using a Bayesian method that explicitly samples unresolved trees[2]. Equal prior probability (0.25) was placed on the three possible resolved trees and the unresolved star tree. To estimate topological bias, recovery of the star tree as the best-supported topology was scored as 1/3 recovery of each resolved phylogeny, and the posterior probability for the star tree was equally distributed among the resolved trees for each replicate.

Literature Cited

1. Yang, Z. & Rannala, B. Branch-length prior influences Bayesian posterior probability of phylogeny. *Syst. Biol.* **54**, 455–470 (2005).

2. Lewis, P. O., Holder, M. T. & Holsinger, K. E. Polytomies and Bayesian phylogenetic inference. *Syst. Biol.* **54**, 241–253 (2005).
